# A practical guide to implementing a successful social media recruitment strategy: lessons from the Eczema Monitoring Online trial

**DOI:** 10.1186/s13063-022-06839-z

**Published:** 2022-10-27

**Authors:** Arabella Baker, Eleanor J. Mitchell, Kim S. Thomas

**Affiliations:** 1 Centre of Evidence Based Dermatology, School of Medicine, University of Nottingham, Applied Health Research Building, University Park, Nottingham, NG7 2RD UK; 2 Nottingham Clinical Trials Unit, School of Medicine, University of Nottingham, Applied Health Research Building, University Park, Nottingham, NG7 2RD UK

**Keywords:** Atopic dermatitis, Eczema, Online recruitment, Randomised controlled trial, Social media, Facebook, Instagram, Internet research

## Abstract

**Background:**

Participant recruitment into clinical trials remains challenging. The global increase in the number of social media users has accelerated the use of social media as a modality of recruitment, particularly during the COVID-19 pandemic when traditional recruitment methods were reduced. However, there is limited evidence on the performance of social media recruitment strategies into eczema clinical trials.

**Methods:**

From September 2021 to January 2022, we recruited participants with eczema into an online randomised controlled trial using free advertising on Twitter, Facebook, Instagram and Reddit (unpaid methods), followed by paid Facebook advertisements (paid method). Unpaid methods were used periodically for 63 days, whilst the paid method for 16 days. Interested individuals who clicked on the advertisement link were directed to the study website, where they could sign up to participate. Consenting, randomisation and data collection occurred exclusively online, using a database management web platform. Evaluation of the social media recruitment methods was performed, including the number of expression of interests, enrolment yield, cost, baseline characteristics and retention.

**Results:**

Our multi-platform based social media recruitment strategy resulted in 400 expressions of interests, leading to 296 participants. Unpaid methods accounted for 136 (45.9%) of participants, incurring no financial cost. Paid Facebook adverts reached 154,370 individuals, resulting in 123 (41.6%) trial participants for a total cost of £259.93 (£2.11 per participant) and other recruitment methods resulted in 37 (12.5%) enrolments. Paid advertisements predominantly attracted younger participants below the age of 20, whereas unpaid methods mainly drew in participants between 20–29 years of age. The social media platforms recruited an ethnically diverse participant population. Completion rate of follow-up was slightly higher for the paid method (*n* = 103, 83.7%) compared with the unpaid methods (*n* = 111, 81.6%).

**Conclusions:**

Unpaid social media posts recruited the most participants; however, it was time consuming for the researcher. Paid Facebook adverts rapidly recruited a large number of participants for a low cost and provided flexibility to target specific audiences. Our findings indicate that social media is an efficient tool that can potentially support recruitment to clinical trials.

**Trial registration:**

ISRCTN45167024. Registered on 29 June 2021.

## Background

Recruitment of participants is crucial for the success of randomised controlled trials (RCTs), but it remains an ongoing challenge [1, 2]. In many RCTs, regardless of specialty, difficulties with recruiting participants and inadequate rates are often reported [3]. A review of 114 publicly funded trials in the United Kingdom (UK) found that only 31% achieved the target sample size [4]. Sully et al. [5] reported that 45% of trials failed to meet recruitment goals and required a time extension. Insufficient recruitment can increase the cost and length of the trial, leading to significant delays or termination of trials [6, 7]. Failure to achieve the target sample size reduces the statistical power of the trial to accurately detect the true effect of the intervention [8]. Poor recruitment may cause scientific, economic and ethical implications and create research waste [9].

Participant recruitment for RCTs often relies on clinician referrals and the performance of recruiting teams. Traditional recruitment methods include: approaching individuals in clinic, via mail or telephone by using health records, newspaper advertisements, posters, flyers, radio and television appearances [10]. However, the increased number of internet users worldwide and the emergence of the global coronavirus (COVID-19) pandemic has accelerated the use of social media as a modality of recruitment [11]. In this paper, social media is defined as websites and internet-based applications that enable the creation, distribution and exchange of user generated content and interaction with fellow users. Social media has a potential for broad reach and capacity to target specific audiences, making it a potentially impactful advertising channel.

Despite the upsurge of social media recruitment strategies, adequate and comprehensive evaluation of these methods is scarce [12]. Studies evaluating RCT recruitment methods have focused on comparing traditional strategies with social media strategies [13–15]. However, studies have rarely utilised unpaid and paid social media recruitment methods simultaneously and little research has concurrently evaluated and compared the performance and cost implications of recruiting on different social media platforms. Evaluating approaches of recruitment to clinical trials has been highlighted as a priority topic for trial methodology research [16].

In this paper, we describe our successful social media recruitment strategy using both unpaid and paid methods for an online eczema RCT. We aim to contribute to the evidence base for running social media recruitment campaigns efficiently and provide a practical “How to guide” for researchers considering the use of social media for participant recruitment.

## Methods

### Study overview

The Eczema Monitoring Online (EMO) study is an RCT, assessing the effect of regular symptom monitoring on eczema severity, conducted entirely online. The trial was prospectively registered on the ISRCTN registry on 29 June 2021 (reference number: ISRCTN45167024). The target population was parents/carers of children with eczema and young people and adults with eczema. Electronic patient-reported outcome measures (ePROMs) were used throughout the study. In this methodological trial, the intervention was online eczema questionnaires. Participants received either a weekly hyperlink to a questionnaire to complete for 8 weeks (intervention group) or only at week 8 (control group). The detailed trial outline is described in the EMO trial protocol, which was made publicly available on 13 August 2021 [17]. Prior to recruitment, ethical approval for conducting the EMO trial was obtained from the University of Nottingham. As part of our ethics application, we submitted examples of texts and images that we were planning to use to advertise the study, but noted that content of the adverts was likely to be slightly adapted during the campaign to suit the different requirements of the social media platforms and tailor the content to the target audience. In this paper, we evaluate the performance of the social media recruitment methods used in the EMO trial.

### Data collection and enrolment

Recruitment, consenting, randomisation and data collection was undertaken exclusively online using the Research Electronic Data Capture (REDCap), a secure database management web platform [18]. Trial-related activities, including recruitment, were managed by the lead researcher who completed this low budget trial as part of a PhD project. Recruitment occurred between 14 September 2021 and 16 January 2022 (4 months), using various social media platforms (described below) for advertising. Individuals who clicked on the study advertisement link were directed to the study website at www.emostudy.org [19], which outlined the aims of the study, eligibility criteria and full participant information. Interested individuals signed up via the study website. Once the electronic consent form was signed, participants completed eligibility checks and were randomised. Randomisation was performed in REDCap, which contains a web-based built in randomisation system. The randomisation schedule was based on computer generated random permuted blocks of randomly varying size, stratified by baseline disease severity and age. Enrolled participants were sent an automated welcome email, based on their group allocation, immediately after enrolment explaining what happens next. Upon completion of the follow-up questionnaire, participants had the opportunity to be entered into a prize draw for a chance to win one of six Amazon vouchers worth £20 each.

### Recruitment strategies

We aimed to enhance recruitment efficiency and minimise cost by utilising an extensive social media advertising campaign that employed various social media platforms simultaneously. This social media-based approach was augmented by using both unpaid and paid recruitment methods. Unpaid methods refer to adverts displayed on social media that did not require any monetary contribution to share with users, whereas the paid method denotes adverts that incurred financial costs to run the adverts and reach users. For unpaid advertising of the study Reddit, Instagram, Facebook and Twitter social media platforms were used, incurring no direct advertisement costs. In addition, paid advertising was used on Facebook. The goal was to maximise the reach of different demographics by utilising a range of social media outlets and implementing a recruitment strategy that took advantage of the different forms of content sharing avenues on each platform, including: hashtags (categorises keywords to help discovery of content by users interested in the topic), following relevant organisations, tagging of followers (alerts users about updates), “Stories” (visual mode of content sharing of user-generated images/videos that disappear after 24 h) and “Reels” (allows the creation of short videos using pre-existing sound clips). Of note, some of our participants learnt about the study by other recruitment modes that did not involve social media, including word of mouth, web search, participant recruitment website, NHS website, poster, Mumsnet and email. Since this paper is concerned with the performance of social media recruitment methods, other modalities of recruitment will not be assessed and discussed in detail.

### Free advertising on social media platforms (unpaid methods)

Unpaid recruitment methods were used periodically for 63 days from 14 September to 18 November 2021. Content was produced by the lead researcher, using a freely available graphic design software [20] and free images [21]. At the design stage of the trial, six patient and public involvement (PPI) panel members provided feedback on some of the social media advertising materials.

#### Instagram

Instagram is a photo and video sharing social media platform with over 1.3 billion users [22]. Prior to study launch, a study specific Instagram account was set up. To build anticipation for the start of recruitment, three countdown posts indicating the number of days until the launch of the study, and a “Stories” post were shared. In addition, 3 days before the study opened to recruitment, a 30-s video of the lead researcher talking about the study and a longer 51-s video with the same concept were released 1 day after study launch. During the recruitment period, altogether, three written posts, one “Reels” post (1-s video clip) and two “Stories” posts, were shared. In all forms of study publicity on Instagram, relevant hashtags (#eczema, #eczemahelp, #eczemasupport, #eczemaresearch) were used to help reach the target audience.

#### Twitter

*Twitter* is a social networking site with 436 million users, where individuals communicate in short messages called *tweets* with a maximum character limit of 280 [22]. Before the study went live, a Twitter account for the study was created using the study name and logo. To raise awareness about the study and build an online network for advertising the study, organisations and charities affiliated with eczema and skin research were followed. Individuals were also followed if they were open about having eczema or being an eczema advocate. In anticipation for the study launch, four countdown tweets were shared. One day before the beginning of recruitment, the existing 30-s video was shared on this platform too. A total of seven tweets were created using hashtags and sometimes the tagging function to add relevant organisations that might reshare the tweet and help to reach the target audience.

#### Facebook

Facebook is social media network that enables users to share content and keep in touch with other users. Facebook is the most widely used social media platform worldwide with over 2.9 billion users [22]. A Facebook page was created for the study with the use of the study logo. This page provided information about the study and contained the address of the University of Nottingham to build credibility with potential participants. The lead researcher interacted with eczema organisations by “liking” their pages. Altogether, four posts were shared prior to study launch, followed by four recruitment posts.

#### Reddit

Reddit is a social media platform that has 430 million users [23]. Reddit consists of a large collection of online forums divided by topics where users can share, rate and comment on content. An account for the study was created and the lead researcher joined different forums (called subreddits) to advertise for recruitment, including eczema support groups, various local and regional cities and towns. For enhancing geographical coverage and representativeness, posting of adverts also occurred in the subreddits of the four UK countries.

### Targeted paid advertising on Facebook (paid method)

Targeted paid advertising on Facebook was used for 16 days from 28 December 2021 to 16 January 2022. Facebook was selected for paid advertisement, as opposed to search engine adverts, due to its optimisation capabilities allowing for targeted advertising, flexible scaling of advert spend and advanced tracking of advert performance. The paid Facebook adverts ran separately from the rest of the social media recruitment campaign. Since Facebook owns Instagram, this configuration allowed us to concurrently recruit participants from both platforms through the paid Facebook adverts. To initiate the use of Facebook advertising, we “boosted” an existing post (boosted post 1). Boosted posts are the simplest form of paid adverts, whereby money is applied to an already present post on the Facebook page to reach a specified audience within a chosen budget and timeline [24]. Although a boosted post has limited customisation features, it allowed us to enhance visibility and its simplicity made it an ideal tool for piloting the paid strategy and determine its feasibility, warranting the use of subsequent paid adverts. To avoid imbalance in age groups, we targeted varying ages with the paid adverts. Since boosted post 1 was more likely to appeal to a younger audience, we targeted the 15–30 age groups. Another existing post was also boosted (boosted post 2), targeting individuals 18 years and above.

The Facebook Ads Manager advertisement management platform was used to create two paid targeted adverts. The advanced customisation features in the Ads Manager allowed the adverts to be specific and tailored based on: goal (e.g. links clicks or increase the number of website visitors), targeted audience (e.g. age, gender, location), allocated budget and duration of the advert. The selection of automatic placements option enabled the adverts to be displayed across interconnected platforms, such as Instagram and Messenger. The aim of this strategy was to achieve broad reach, various demographics and optimise link clicks by individuals interested in taking part. The Ads Manager facilitated the monitoring of advert performance. While the targeted paid adverts were running, performance statistics were reviewed regularly and spending limits were modified according to the success of the individual advertisements.

**Fig. 1:**
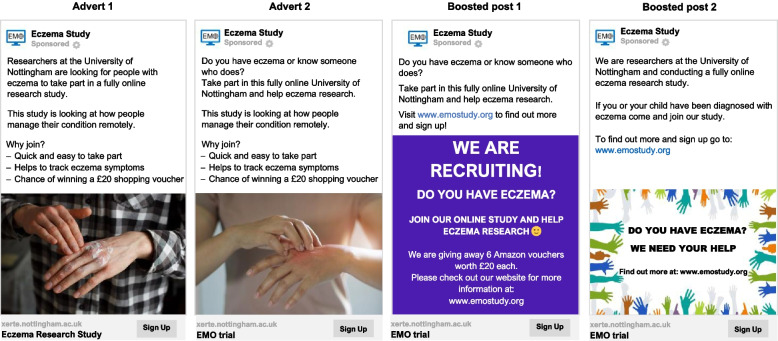
Screenshot of adverts and boosted posts used in paid Facebook advertisements

The first advert was created with the goal of increasing website visitors. The automatic advert placement option was utilised, enabling the dissemination of the advert to a wide and potentially eligible population. The target audience initially consisted of people aged 14 years and above, any gender and living in the UK and Ireland. After 4 days, the audience of this advert was altered to specifically target men only to try to prevent significant gender imbalance that started to occur in the trial. After 3 days, the target audience was reset to the demographics of the original advert, except for age which was raised to 16 years and above. The first Facebook advert ran periodically between 28 December 2021 and 16 January 2022 for 16 days in total. The second advert was set up using a similar strategy to the first advert that included any gender; however, the location of the target audience differed to enhance ethnic diversity. Individuals from Birmingham (+ 40 km) and London (+ 40 km), where the population of ethnic minority groups is higher, were specifically targeted alongside the four UK countries. The second advert ran between 11 and 16 January 2022 (6 days). Examples of Facebook adverts used for recruitment can be found in Fig. 1.

### Analysis

The performance of the unpaid and paid methods was assessed by calculating enrolment yield, defined as the proportion of enrolled participants out of those who expressed interest in the trial but did not reach enrolment. Descriptive statistics were used to report the baseline characteristics of participants, including age, gender, ethnicity and country of residence. The number of recruited participants was plotted by displaying the weekly enrolment rates of unpaid methods alongside the paid method throughout the study period. Retention was assessed by calculating the number of participants who completed follow-up according to recruitment method.

The success of Facebook adverts was evaluated via the Facebook Ads Manager application that autogenerated metrics of engagement activity, providing a summary of the performance and cost of individual adverts. Measures for analysis included the following: (1) reach, which describes the number of people who saw the advert at least once; (2) link clicks, which indicates the number of clicks on the link displayed in the advert; (3) cost per link click, which refers to the average cost for each link click; and (4) recruitment cost per participant, which is calculated by dividing advertising costs with the total number of enrolled participants. The direct advertising cost of each recruitment method was recorded. Although staffing time and costs were not tracked, approximately 5 to 7 hrs per week was spent on the unpaid recruitment methods that involved written and visual content creation specific for the different social media platforms, posting in forums, dealing with queries of potential participants and reacting to comments. Operating paid Facebook adverts and tracking performance required approximately 4 hrs per week. The indirect cost of staff who are working on managing the social media campaign depends on their hourly rate or wage; therefore, staffing cost can vary greatly based on position of employment and needs to be estimated accordingly.

## Results

Altogether, 400 expressions of interests were recorded during the study. Over a four-month period, a total of 296 participants were enrolled into the study (Table 1). Unpaid social media recruitment methods accounted for 136 (45.9%) of trial participants, paid methods for 123 (41.6%) and other methods of recruitment resulted in 37 (12.5%) enrolments.

**Table 1:** Number of expression of interest and enrolled participants using unpaid, paid and other methods of recruitment during the trial

Recruitment type	Number of expression of interest	Enrolment yield, *n* (%)
**Paid method**		
Facebook	55	41 (75)
Instagram	122	82 (67.2)
**Total of paid method**	177	123 (69.5)
**Unpaid methods**		
Reddit	152	121 (79.6)
Twitter	11	7 (63.6)
Facebook	4	2 (50)
Instagram	8	6 (75)
**Total of unpaid methods**	175	136 (77.7)
**Other methods**		
Word of mouth	19	14 (73.6)
Participant recruitment website	9	8 (88.8)
Poster	2	2 (100)
East Midlands PGR conference	1	0
Web search	8	5 (62.5)
Mumsnet	1	1 (100)
Email	1	1 (100)
NHS website	6	6 (100)
Unknown	1	0
**Total of other methods**	48	37 (77)

*n,* enrolled participants; *%*, percent enrolled out of those who expressed interest in the study

**Fig. 2:**
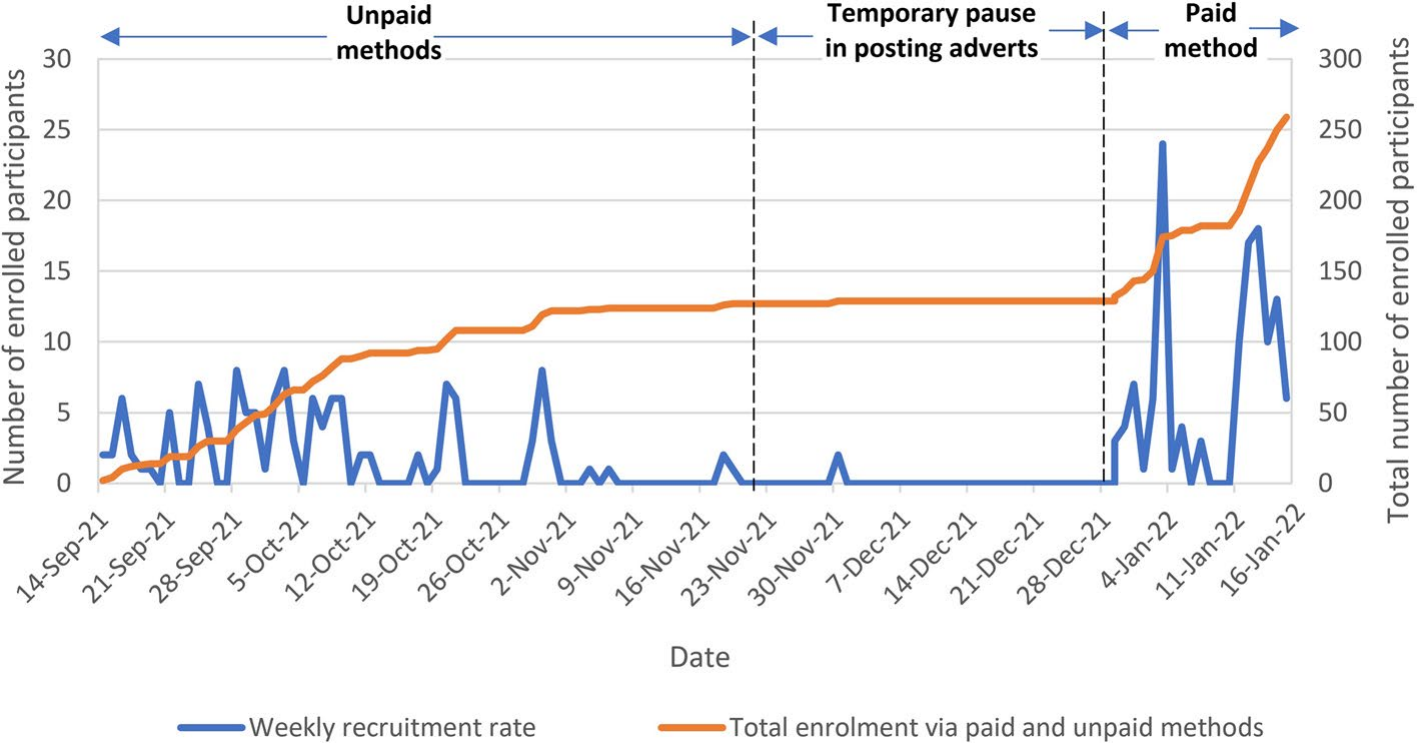
Weekly recruitment rate of unpaid and paid methods

The number of recruited participants per day differed across the recruitment methods, the highest number of participants from unpaid methods (*n* = 9) joined the study on 28 September 2021 and from paid Facebook adverts (*n* = 25) on 3 January 2022. Differences in recruitment rate by the unpaid and paid methods can be noted throughout the study, which affected the overall recruitment rate as shown in Fig. 2. Reasons for these fluctuations include periodic advertising via the unpaid methods and modifications to paid Facebook adverts that was underpinned by intermittent pauses in advertising. Advertising breaks could have affected recruitment but proved to be useful, allowing to observe and evaluate the effect of temporary pauses of advertisements on recruitment rate.

In order to gauge information on recruitment timeline trends, the sign-up date and time of each participant by recruitment platform was tracked throughout the study. Most of our participants, regardless of recruitment platform, signed up in the evening after 5 PM (*n* = 138, 46.8%), especially during weekdays. Weekends and particularly Friday evenings usually generated increased traffic for the adverts, which in turn enhanced recruitment. Completion rate of follow-up was slightly higher for the paid method (*n* = 103, 83.7%) compared with the unpaid method (*n* = 111, 81.6%).

### Cost and performance of paid Facebook advertisements

During a brief paid advertisement period of 16 days on Facebook, 123 participants were recruited for a total cost of £259.93. The average cost per link click reported at £0.14 and the overall cost per enrolled participant arising from the paid advertisements was £2.11. Table 2 summarises the performance and itemised cost of each paid targeted advert on Facebook. The aggregated reach of the four Facebook advertisements was 154,370 individuals. Most adverts were placed on Facebook by default, reaching 94,096 individuals, whereas adverts displayed on Instagram reached 60,274 individuals. Even though paid adverts on Instagram reached fewer people, twice as many participants were recruited from Instagram (*n* = 82), compared to Facebook (*n* = 41).

**Table 2:** Summary of performance of paid targeted adverts on Facebook

Modality	Duration	Reach	Link clicks	Cost per link click	Advert cost
Advert 1	16 days	93,630	1,128	£0.16	£176.94
Advert 2	6 days	33,035	353	£0.17	£59.99
Boosted post 1	13 days	24,637	306	£0.06	£18.00
Boosted post 2	2 days	3,068	34	£0.15	£5.00
Total	^a^	154,370	1,821	£0.14	£259.93

^a^Data not available due to adverts running concurrently

### Participant characteristics

Participants from diverse demographic backgrounds were recruited through social media (Table 3). The age of participants ranged from 2 to 74 years. Most of the enrolled participants were young, aged 14–19 years (35.1%) and were recruited mainly by paid Facebook adverts displayed on Instagram (*n* = 82). In contrast, those aged 20–29 (30.4%) primarily joined the trial from the unpaid method of Reddit (*n* = 67), while most participants 50 years old and above (9.3%) enrolled primarily via paid Facebook adverts (*n* = 23). Thus, paid advertisements predominantly attracted younger participants below the age of 20, whereas unpaid methods mainly drew in participants between 20 and 29 years of age (Table 3). Unexpectedly, very poor recruitment of parents of children with eczema occurred (*n* = 15). The social media platforms recruited an ethnically diverse participant population as shown in Table 3.

**Table 3:** Baseline participant demographics and self-reported method of recruitment

Characteristic	Total, *n* (%)	Reddit	Facebook	Instagram	Twitter	Other^a^
**Age range (years), *n* (%)**						
0–13	15 (4.9%)	2 (0.6%)	2 (0.6%)	1 (0.3%)	4 (1.4%)	6 (2%)
14–19	104 (35.1%)	14 (4.7%)	2 (0.7%)	81 (27.4%)	0	7 (2.3%)
20–29	90 (30.4%)	67 (22.6%)	3 (1%)	4 (1.4%)	1 (0.3%)	15 (5.1%)
30–39	43 (15%)	30 (10.1%)	7 (2.4%)	2 (0.7%)	0	4 (1.3%)
40–49	16 (5.4%)	5 (1.7%)	6 (2%)	0	2 (0.7%)	3 (1%)
50–59	13 (4.3%)	3 (1%)	9 (3%)	0	0	1 (0.3%)
60–69	10 (3.3%)	0	9 (3%)	0	0	1 (0.3%)
70–74	5 (1.6%)	0	5 (1.6%)	0	0	0
**Ethnicity, n (%)**						
White	228 (77%)	92 (31.1%)	41 (13.9%)	57 (19.3%)	7 (2.3%)	31 (10.4%)
Asian	36 (12.1%)	19 (6.4%)	1 (0.3%)	13 (4.4%)	0	3 (1%)
Mixed background	15 (5.1%)	8 (2.7%)	0	6 (2%)	0	1 (0.3%)
Black	13 (4.4%)	0	0	11 (3.7%)	0	2 (0.7%)
Another ethnic group	4 (1.4%)	2 (0.7%)	1 (0.3%)	1 (0.3%)	0	0
**Gender, n (%)**						
Male	77 (26%)	49 (16.6%)	11 (3.7%)	6 (2%)	2 (0.7%)	9 (3%)
Female	210 (71%)	69 (23.3%)	32 (10.8%)	76 (25.7%)	5 (1.7%)	28 (9.5%)
Other	3 (1%)	2 (0.7%)	0	1 (0.3%)	0	0
Prefer not to say	6 (2%)	1 (0.3%)	0	5 (1.7%)	0	0

^a^Includes word of mouth, web search, participant recruitment website, NHS website, Mumsnet, poster and email

Given that this was a fully online eczema trial, there was no restriction on geographical location of participants. The recruitment strategy broadly focused on the UK, but individuals residing in other countries were able to join the study if they were eligible. However, the paid Facebook adverts targeted only the UK, Isle of Man and Ireland. The geographical reach within the UK was noteworthy; enrolments occurred from all four UK countries. Most enrolled participants were from England (*n* = 181), with the remaining residing in Scotland (*n* = 23), Northern Ireland (*n* = 16) or Wales (*n* = 14). Sixty-two participants (21%) joined the study from 16 other, mainly English speaking countries, such as Isle of Man (*n* = 17), USA (*n* = 16), Ireland (*n* = 13), Australia (*n* = 2) and Canada (*n* = 1). Due to its international coverage, Reddit recruited most non-UK residing participants out of all social media platforms. When it comes to social media recruitment, exploring the attributes of the different platforms can aid the selection of the appropriate advertising outlet. Table 4 summarises the advantages and disadvantages of the social media platforms we used to advertise.

**Table 4:** Advantages and disadvantages of social media platforms used for advertising

Platform	Advantages	Disadvantages
Facebook (paid)	• Most social media users • User friendly • Interconnected with other platforms • Wide reach • Demographic targeting • Custom audiences • Performance tracking • Optimising capabilities	• Cost based on link clicks not on actual enrolments • Approval of advert may take 24 hrs • Advert may be rejected by moderator • Digital skills required to craft a well performing advert • Adverts can be fatigued • Decreasing popularity with users
Reddit (unpaid)	• Simple to use • Diverse user base • Posting in forums is free • UK and international coverage	• Post reactive platform • Overflowing content in subreddits • Visibility of post decreases quickly • Requires regular posting • Time-consuming for researcher • Knowledge of Reddit-specific terminology is needed
Twitter (unpaid)	• Often used for recruitment • Free to post • Hashtags help the discovery of the posts by users interested in the topic	• Limited character count • Shorter content is needed • Reduced freedom in content creation • Poor organic reach • Time-consuming for researcher
Instagram (unpaid)	• Popular platform • A lot of active users • Free to post • Appealing interface • Organised layout of posts • Many creative and fun features for creating posts (emoji, music, filters) • Various content sharing formats (images, videos, short sound clips)	• Cannot target specific audiences • Poor organic reach • Only optimised for app use, its web-version is substandard • Requires capturing content • Limited insight into performance of posts • Creating different types of content formats can be time-consuming
Facebook (unpaid)	• Creation of study specific Facebook page, instead of profile, increases credibility • Free to post	• Difficult to gain followers • Poor organic reach • Cannot target specific audiences • Many features only available when paying for the adverts

## Discussion

The purpose of this paper was to evaluate the performance of social media recruitment, using both unpaid and paid methods for an eczema RCT. The unpaid methods recruited the most participants, resulting in nearly half of total enrolments. These methods did not incur any advertising costs and recruited a diverse study population. Paid adverts on Facebook rapidly recruited participants for a total cost of £259.93 (£2.11 per participant). This cost is significantly lower than reported in previous eczema recruitment studies with a total spending of US$10,064 for an online feasibility study [25] and cost per participant of AUD$ 2494 for a single-centre RCT [26]. The latter study reported major challenges in recruitment, despite employing two recruitment agencies for running the social media advertising campaigns. Of note, employing recruitment agencies has high cost implications, yet may not yield sufficient enrolments.

Since our recruitment strategy aimed to minimise cost, the lead researcher fully managed the social media campaign throughout the trial by adopting an autodidact approach to learning the specifics of each platform and actively searching for free advertising opportunities on social media. Thus, our remarkably low recruitment spending was partly due to identifying a social media platform (Reddit) that allowed free posting in forums. The use of Reddit helped to preserve the study budget and added novelty to the recruitment strategy as it has not been used for eczema RCTs. Our results demonstrated the feasibility of unpaid Reddit adverts in reaching a considerable sample of participants for free (*n* = 121, 40.8%), enhancing demographic diversity with no cost implications. These findings resonate with an online psychology study that successfully recruited participants through this platform [27].

The success of our recruitment strategy might have been partially related to the fact that eczema is a prevalent chronic skin condition [28] and people with eczema often search online for advice about the management of the condition [29]. Despite eczema being common in childhood, we only managed to recruit 15 parents of children with eczema. This exceptionally low recruitment rate of this population was unanticipated, and it contradicts with other eczema studies that successfully recruited this demographic from social media [30, 31]. The reason for the low presence of children is not clear but might be related to the methodological nature of this RCT in which the intervention was online questionnaires rather than a treatment intervention that might have decreased the interest of busy parents to take part.

Female participants were overrepresented, by almost threefold. However, this gender imbalance commonly occurs in other eczema studies too [30, 32] and might be related to gender differences in internet use whereby females are more likely to use the internet to communicate and exchange information, whereas males prefer to browse and seek information on the internet [33].

Interestingly, there was a profound difference in the recruitment pace of the unpaid methods and the paid method (Fig. 2). In particular, Reddit was a post reactive platform where the number of recruited participants rapidly increased from the point of posting the advert that culminated at 2 days, followed by a drastic decrease and even a halt on recruitment afterwards. As depicted in Fig. 2, when a longer period of break was applied, recruitment practically stopped (between 1 and 28 December 2021). Therefore, this platform requires regular posting of adverts to allow for adequate recruitment. Conversely, paid adverts on Facebook gradually reached potential participants, steadily increasing recruitment stream.

We recommend running paid Facebook adverts for at least 7 days to take advantage of its streamlined algorithm that propagated the advert into the related social media networks to enhance the reach of the target audience. In terms of timing of social media adverts, our findings indicate that for optimal results the adverts ought to be scheduled when people are likely to have spare time, such as upon finishing work and over the weekend. These timeframes provide a good window of opportunity for recruitment.

Regarding advantages of social media recruitment, the unpaid adverts involved no direct payment yet recruited many participants, whilst paid Facebook adverts provided flexibility to target specific audiences; though costs were incurred, a predetermined spending limit was set which could be regularly altered. This pragmatic feature is particularly useful for researchers working within financial constraints. Through unpaid methods, altogether, 136 (45.9%) participants were recruited for free in approximately 2 months, whereas paid Facebook advertisements, for a moderate total cost of £259.99, recruited 123 participants (41.6%) in only 16 days of active recruitment. Enrolment yields in our study were higher in contrast with other dermatology studies that utilised social media to recruit [26, 32], and our recruitment duration was relatively short. These findings are consistent with a recent recruitment study, which found that targeted social media campaigns shortened the duration of participant recruitment into an eczema RCT [34].

Despite the advantages described, recruiting on social media has several drawbacks. Concerns may prevail about digital exclusion when only recruiting via internet-based methods, such as social media. However, according to the Office for National Statistics [35], in 2020, 96% of UK households had internet access. In 2021, 53 million (77.9%) of the UK population were active social media users [36]. A limitation of the unpaid recruitment methods was that they were more resource intensive for researcher time due to having to create posts regularly that were in line with the specific requirements of each platform. For instance, Twitter had a limit of 280 character count, Reddit posts had to be regularly shared to ensure visibility of the advert as content was pushed down due to continuous new posts by other users. Furthermore, unpaid adverts do not allow to target specific audiences. Conversely, creating and navigating paid targeted adverts on Facebook was easier and required less time than the unpaid methods. Prior to an advert going live, it is standard practice for a Facebook moderator to review and approve the advert, including image and text. If either is deemed to be inappropriate, it leads to rejection, thereby the content needs to be altered and resubmitted for review. It is worth noting that any alteration to the existing Facebook advert (budget, target audience) is reassessed by a moderator and during this timeframe the advert is paused. Despite these shortfalls, traditional recruitment methods such as mailing invitations and flyers are often more labour intensive and costly than social media advertising. This was highlighted in several studies comparing social media and traditional methods, indicating that social media recruitment methods are more efficient from a cost and time perspective and help to reach a diverse pool of potential participants, thus aiding generalisability [14, 37, 38]. Our study demonstrates that both unpaid and paid social media recruitment methods represent viable alternatives to reach and enrol participants into eczema RCTs.

A limitation of our study is that the unpaid social media platforms by default failed to provide information on how many individuals were reached by the adverts and how many clicked on the advert link. Facebook produced performance metrics only for paid adverts. This hinders accurate response analysis and comparison of the unpaid and paid advertisements performance on the various social media platforms. In addition, exposure to the advertisement is associated with time spent on social media increasing the potential for selection bias, thereby recruitment can be skewed towards those often using social media. Consequently, regular users of social media were more likely to come across our posts and adverts than those who spent less time on these platforms. Another limitation of our study is that staffing cost and time of developing and monitoring adverts was not tracked, yet it could have provided a more comprehensive analysis. However, this was partly because we performed a post hoc analysis of our recruitment strategy owing to its unexpected success in reaching the target sample size in a short timeframe. Lastly, given that social media recruitment methods were used at different times of the year, with unpaid adverts between September and November 2021 and paid adverts at the end of December 2021 around New Years and in January 2022, direct comparison of the performance of the recruitment methods cannot be made because these timings could have affected uptake. Perhaps, advertising during the festive period might have led to our unexpected finding, namely, that Instagram was more successful in recruiting participants despite having lower reach than Facebook. Presumably, this might have occurred due to different demographics, as each social media platform engages different audiences. Since we were advertising during the Christmas break, it is likely that more young people were online during this time-period compared to school term, which increased their likelihood of coming across our adverts displayed on Instagram and signing up for this study. The prize draw of 6 × £20 Amazon vouchers might also have been a factor in attracting our younger participants.

## Conclusions

Recruitment on social media was successful in recruiting participants with eczema for an online RCT. This paper adds valuable data to the evidence base on the feasibility and performance of social media recruitment campaigns. Our findings provide information on the practicalities and benefits of using social media for recruitment; however, further research is required to establish the efficacy of social media for targeting parents of children with eczema. It would be also beneficial to assess whether the time of the year, especially holidays, affects the recruitment rate of the social media adverts. Continued effort, adequate evaluation and systematic reporting of recruitment strategies is required to enable researchers to select the most appropriate strategies for recruiting participants into RCTs. Social media is a promising tool that has a unique ability to transcend barriers to recruiting participants and potentially revolutionise recruitment to clinical trials.

## Data Availability

The datasets used and/or analysed in this paper are available from the corresponding author on reasonable request.

## Abbreviations

COVID-19: Coronavirus disease
EMO: Eczema Monitoring Online
ePROM: Electronic Patient Reported Outcome Measure
PPI: Patient and public involvement
RCT: Randomised controlled trial
REDCap: Research Electronic Data Capture
UK: United Kingdom
